# Redox Regulation in Glioblastoma: Mechanisms, Biomarkers, and Therapeutic Implications

**DOI:** 10.3390/ijms27177940

**Published:** 2026-09-06

**Authors:** Salma Lamrabet, Asmae Squalli Houssaini, Sanae Bennis, Rabii Ameziane El Hassani

**Affiliations:** 1 Biomedical and Translational Research Laboratory, Faculty of Medicine and Pharmacy, Sidi Mohamed Ben Abdellah University, Fez 30000, Morocco; 2 Laboratory of Biology of Human Pathologies, Faculty of Sciences of Rabat, Mohammed V University, Rabat 10000, Morocco

**Keywords:** glioblastoma, oxidative stress, radioresistance, chemoresistance, tumor heterogeneity

## Abstract

Glioblastoma is the most aggressive primary tumor of the central nervous system, characterized by high invasiveness, rapid progression, and a poor prognosis despite the current treatment modalities. Molecular stratification, using biomarkers such as *IDH1*, *TERT*, and *MGMT*, is a crucial step in the 2021 WHO classification for improving diagnosis and prognosis. Oxidative stress, a feature of GB, has been identified as an important factor in the initiation, progression, and resistance to treatment. It occurs due to an imbalance between reactive oxygen species generated by mitochondrial metabolism, NADPH oxidases, and exogenous sources such as ionizing radiation and xenobiotics and antioxidant defense. This imbalance leads to DNA damage, genomic instability, and deregulation of signaling pathways involved in cell proliferation, apoptosis, and tumor progression. This review provides an overview of key oxidative stress biomarkers and their dual roles in tumor suppression and progression. It highlights how oxidative stress contributes to treatment responses and resistance to current GB treatments, including redox-adaptive mechanisms such as the Nrf2–Keap1 pathway, which promotes radioresistance. Finally, it discusses the potential of understanding these mechanisms to develop therapeutic strategies that target redox balance and homeostasis, aiming to overcome resistance and improve survival outcomes for glioblastoma patients.

## 1. Introduction

Gliomas are the most common primary tumors of the central nervous system (CNS), arising from glial cells, and gliomagenesis is a highly complex process driven by genetic alterations and epigenetic modifications. These mechanisms are implicated in the transformation of normal glial cells, such as astrocytes and oligodendrocytes, into malignant cells capable of intense proliferation, diffuse parenchymal infiltration, and robust angiogenesis [1].

## 2. Methods

To critically evaluate the current body of evidence, this review examines the emerging role of oxidative stress and redox dysregulation in glioblastoma progression, therapeutic resistance, and clinical management. A literature search was conducted using the PubMed/MEDLINE, Scopus, and Web of Science databases. The search focused primarily on articles published between 2020 and 2026, alongside older references included for foundational context. Search strategies employed combinations of keywords and Boolean operators, including “glioblastoma,” “oxidative stress,” “reactive oxygen species,” “redox,” “NRF2,” “KEAP1,” “NOX4,” “biomarkers,” “radioresistance,” “chemoresistance,” and “temozolomide.”

Inclusion criteria comprised original research articles, preclinical (in vitro and in vivo) studies, clinical studies, and relevant literature reviews. Eligible studies focused on oxidative stress mechanisms or biomarkers in glioblastoma; when glioblastoma-specific literature was limited, high-quality studies in other cancers were included as pathway-level comparators. Exclusion criteria consisted of non-peer-reviewed conference abstracts, non-English publications, and case reports. Two authors independently screened titles and abstracts to assess initial eligibility, followed by a full-text review of selected studies. Disagreements regarding study selection were resolved through joint discussion and consensus with the senior authors.

## 3. Molecular and Clinical Features of Glioblastoma

Gliomas include a wide range of malignant cancers, from low-grade diffuse astrocytomas to highly aggressive glioblastoma (GB) (WHO grade 4). The latter is the most common and aggressive primary malignant tumor of the CNS in adults, accounting for approximately 45–50% of all malignant primary brain tumors [2]. It has an age-adjusted annual incidence of 3–5 cases per 100,000 people [3,4]. The peak incidence occurs between 55 and 75 years of age, with strong therapeutic resistance and a notably high rate of recurrence [5].

GB is primarily driven by diffuse parenchymal infiltration and significant cellular and molecular heterogeneity (Table 1). However, younger patients may present with biologically distinct variants caused by specific molecular alterations, such as *IDH1* mutations (Table 1) [6,7]. Despite standard multimodal treatment, which includes maximal safe surgical resection, radiotherapy, and concomitant temozolomide (TMZ) chemotherapy (commonly referred to as the Stupp protocol), median overall survival is limited to 15–20 months, with a 5-year survival rate below 10% [8,9,10]. Current research focuses on novel targeted therapies, immunotherapies, and personalized treatment strategies to improve patient outcomes [11,12].

The diagnosis of these tumors is based on integrating neuroimaging with tissue analysis [13]. Magnetic resonance imaging (MRI) is widely used due to its superior soft-tissue contrast and ability to define tumor extent and infiltrative patterns. Typical high-grade gliomas, including GB, often appear as heterogeneously enhancing lesions with central necrosis and peritumoral edema [14]. A definitive diagnosis requires histopathological analysis of tumor tissue obtained through biopsy or surgical resection. The classical histologic features of GB include nuclear atypia, mitotic activity, microvascular proliferation, and necrosis [1]. Molecular study remains essential in the grading process for different stages of tumor malignancy; however, molecular diagnostics are integrated into standard practice to complement histology by detecting key genetic alterations that refine diagnosis and prognosis (Table 1) [15].

Despite its limitations compared to traditional tissue biopsy, liquid biopsy has made remarkable progress as a complementary technique for the diagnosis and molecular characterization of glioblastoma [16,17]. Unlike surgical specimens, liquid biopsy allows the analysis of biomarkers derived from the tumor, including the analysis of circulating tumor DNA, circulating tumor cells, cell-free RNA, microRNAs, extracellular vesicles, proteins and metabolites, providing real-time information on the genomic, transcriptomic, proteomic and metabolomic landscape of GB [16]. Compared to other biofluids, cerebrospinal fluid has the highest diagnostic sensitivity because it is in direct contact with the tumor, contains higher levels of nucleic acids derived from the tumor compared to plasma, and thus is suitable for detecting clinically relevant abnormalities, including abnormalities in *IDH1/2*, *EGFRvIII*, the *TERT* promoter, *TP53*, *PTEN*, and *MGMT* [17,18]. The blood–brain barrier (BBB), on the other hand, restricts the amount of peripheral blood that contains the released biomarkers, leading to lower sensitivity of assays used for blood measurements, but new, highly sensitive technologies, such as digital PCR and next-generation sequencing, are significantly enhancing blood-based diagnostics [16,18].

The 2021 World Health Organization Classification of Tumors of the Central Nervous System (WHO CNS5) marked a major shift from a purely histology-based diagnosis towards an integrated histomolecular approach. This approach stratified the subcategories using combined morphological and molecular criteria, enhancing diagnostic precision and prognostic relevance [19]. According to this classification, the use of “glioblastoma” is reserved for IDH-wildtype astrocytic tumors in adults, even in the absence of high-grade histological features, and in the presence of a *TERT* promoter mutation, *EGFR* amplification, or +7/−10 chromosome modifications (Table 1). On the other hand, patients with the IDH-mutant form are referred to as “astrocytoma grade 4” [7].

Several biomarkers play central roles in GB pathogenesis and clinical management:

**Table 1:** Clinically relevant biomarkers in glioblastoma: Implications for diagnosis and treatment.

Biomarker	Diagnostic/Prognostic Importance	Therapeutic Implications	Key References
IDH1/IDH2	IDH mutations define a distinct subgroup of diffuse gliomas with significantly better prognosis. Adult glioblastoma is defined as IDH-wildtype in the WHO CNS5 classification.	IDH-mutant tumors may benefit from IDH inhibitors (e.g., ivosidenib, vorasidenib).	[6,20]
TERT promoter	TERT promoter mutations promote telomerase activation and are common in IDH-wildtype GB. They are used as molecular diagnostic criteria for GB.	No direct targeted therapy is currently approved. TERT status may influence tumor aggressiveness and sensitivity to experimental telomerase inhibitors and DNA damage-inducing strategies.	[7,21]
EGFR	EGFR amplification and mutations (e.g., EGFRvIII) drive proliferation and invasion and are defining features of GB in IDH-wildtype tumors.	Target of EGFR inhibitors, monoclonal antibodies, vaccines, and CAR-T approaches; clinical efficacy remains limited due to tumor heterogeneity and resistance mechanisms.	[7,22]
TP53 (p53)	TP53 mutations disrupt cell-cycle control and DNA damage response, contributing to genomic instability and tumor progression.	p53 status influences response to radiotherapy and chemotherapy; restoration of p53 signaling and synthetic lethality strategies are under investigation.	[23,24]
ATRX	Loss of ATRX expression is characteristic of IDH-mutant astrocytomas and associated with alternative lengthening of telomeres (ALT).	ATRX loss may sensitize tumors to DNA-damaging agents and ATR/CHK1 inhibitors, potentially exposing vulnerabilities in redox and replication stress pathways.	[7,25]
MGMT promoter methylation	MGMT methylation predicts improved survival and better response to alkylating agents in GB. It is a strong predictive biomarker.	Patients with MGMT-methylated tumors derive greater benefit from temozolomide chemotherapy; unmethylated tumors may require alternative or intensified strategies.	[26]
Loss of heterozygosity 1p/19q	Combined 1p/19q codeletion defines oligodendroglioma and excludes GB diagnosis. Associated with a favorable prognosis.	Predicts sensitivity to alkylating chemotherapy and radiotherapy; not applicable as a therapeutic marker in GB.	[7,27]
CDKN2A/B deletion	Homozygous deletion is associated with aggressive behavior, higher grade, and poor prognosis. It is a grading criterion in diffuse gliomas.	Loss of CDKN2A suggests potential sensitivity to CDK4/6 inhibitors, though clinical efficacy in GB remains under investigation.	[7,28]

Although molecular biomarkers have essentially improved the diagnosis and molecular classification of GB, they do not fully elucidate the mechanisms underlying its aggressive behavior and therapeutic resistance [6,29,30]. Increasing evidence indicates that metabolic reprogramming and redox homeostasis disruption are fundamental elements of GB pathogenesis [31,32]. Elevated reactive oxygen species (ROS), generated through mitochondrial dysfunction, oncogenic signaling, hypoxia, and NADPH oxidase activation, promote genomic instability while simultaneously activating signaling pathways that support tumor proliferation, angiogenesis, invasion, stemness, and resistance to radiotherapy and TMZ [31,33]. Consequently, redox-associated biomarkers have emerged as promising complementary indicators for prognostic assessment and therapeutic targeting in GB. The following sections discuss the principal oxidative stress biomarkers and their clinical relevance in glioblastoma.

## 4. Redox Signaling Networks in Glioblastoma

### 4.1. Endogenous Cellular Generation of ROS

Oxidative stress and the antioxidant balance (redox balance) are widely discussed topics implicated in the development and progression of several diseases, including atherosclerosis, diabetes mellitus, stroke, and cancer [34]. Reactive oxygen species are generated during both physiological and pathophysiological signal transduction, originating from multiple cellular organelles, including the cytoplasm, endoplasmic reticulum, mitochondria, and peroxisomes, as part of the cell’s basal metabolic function [35]. The accumulation of ROS in the intracellular compartment has been associated with dysfunction in various signaling pathways, including disruptions in gene expression, apoptosis, and cell proliferation [36,37]. Key signaling pathways disrupted include NF-κB, RAS/RAF, and MAPK1/2, which are related to the expression of a mutated *TP53* tumor suppressor gene [35].

Damage has also been done to proteins and DNA by inducing somatic mutations that affect cancer progression related to genomic instability and cell dysregulation [34,35].

#### 4.1.1. Biomarkers Related to Mitochondrial Oxidative Metabolism

- Mitochondrial Dysfunction, Reactive Oxygen Species, and the Warburg Effect in Glioblastoma

Mitochondria are organelles found in eukaryotic cells; their main roles are to maintain a continuous cellular energy supply and to regulate redox homeostasis. Adenosine triphosphate (ATP), the primary form of energy used by cells, is generated via multiple oxidations in the inner mitochondrial membrane, particularly through the mitochondrial electron transport chain (ETC). During this process, the cell generates a large amount of ROS [38]. These oxidative radicals are mainly generated by mechanisms involving mitochondrial complexes, a series of five multi-subunit complexes (Complexes I–V) embedded in the inner mitochondrial membrane. Electrons generated from the oxidation of metabolic substrates enter the ETC via ubiquinone oxidoreductase (Complex I) or succinate dehydrogenase (Complex II) and are transferred sequentially through ubiquinone (Q coenzyme), cytochrome bc1 (Complex III), cytochrome c, and cytochrome c oxidase (Complex IV), where molecular oxygen is reduced to water [39,40]. The energy released during electron transport drives proton translocation across the inner mitochondrial membrane by Complexes I, III, and IV, establishing an electrochemical gradient that fuels ATP synthesis by ATP synthase (Complex V). Complex I is the key point of entry for nicotinamide adenine dinucleotide (NADH) as an electron donor into the respiratory chain, leading to the release of protons and electrons. However, the elevated concentration of NADH/NAD+ in the mitochondrial matrix produces superoxide anions [41]. They are primarily generated as byproducts of aerobic respiration.

On the other hand, under cellular homeostasis, Complex III also contributes to ROS production by linking the Q coenzyme to cytochrome c. At low levels, superoxide predominantly diffuses into the intermembrane space and the matrix, acting as a signaling molecule in processes such as apoptosis, autophagy, and mitochondrial biogenesis. However, excess superoxide anion induces oxidative stress, which can damage mitochondria, further increasing ROS production and impairing energy generation [41,42].

Succinate dehydrogenase is a unique enzyme in the ETC since it does not participate in proton translocation across the inner mitochondrial membrane. Furthermore, it is a key enzyme that catalyzes the oxidation of succinate to fumarate while simultaneously reducing the flavin adenine dinucleotide (FAD) cofactor to FADH_2_ [40]. Unlike other citric acid cycle (Krebs cycle) enzymes, Complex II is enclosed in the inner mitochondrial membrane, allowing the electrons generated during succinate oxidation to be transferred through a series of iron–sulfur (Fe–S) clusters to Q coenzyme, producing ubiquinol (QH_2_), which subsequently donates electrons to Complex III of the electron transport chain [43]. ROS are produced by Complex II when the mitochondrial membrane potential is higher than normal or when the NADH/NAD+ ratio is elevated; under these conditions, electrons can flow in reverse through Complex II. This reversion can lead to reduced FAD levels and the generation of ROS [42,43].

As discussed previously, ROS are primarily produced under physiological and pathophysiological conditions [44]. However, their influence on glioma development remains under investigation. The major cellular source of oxygen radicals in glioblastoma could be associated with the ETC [45,46]. This delicate redox balance is disrupted, resulting in persistently elevated ROS levels that promote tumor growth while remaining below the threshold that would induce extensive oxidative damage. Consequently, GB cells exist in a state of chronic oxidative stress that supports tumor progression and therapeutic resistance rather than apoptosis [47,48].

Certainly, cancer cells exploit mitochondrial structural abnormalities to support their malignancy; for instance, the ETC is inefficient due to multiple electron-leakage sites. Thus, the complexes react with molecular oxygen to form superoxide [49]. Mitochondria are a principal endogenous source of ROS in glioblastoma. However, structural and functional abnormalities frequently observed in GB mitochondria, including altered mitochondrial DNA, dysregulated respiratory complexes, and impaired mitochondrial dynamics, increase electron leakage, particularly from Complexes I and III [50,51,52]. These escaped electrons react prematurely with molecular oxygen to generate superoxide anions, which are subsequently converted to hydrogen peroxide by mitochondrial superoxide dismutase [53,54]. Although hydrogen peroxide is less reactive than superoxide, it diffuses smoothly through cellular compartments and functions as an important second messenger regulating signaling pathways involved in proliferation, angiogenesis, invasion, and survival [55].

Beyond serving as a source of ROS, mitochondria are now recognized as dynamic signaling organelles that regulate apoptosis, calcium homeostasis, biosynthesis, and metabolic adaptation [56,57].

Glial cells show remarkable plasticity, with variable mitochondrial morphology and altered cellular respiration in response to multiple variations in oxygen and glucose supply under therapeutic stress [53]. Mitochondrial ROS (mtROS) activate several oncogenic signaling pathways, including PI3K/AKT, NF-κB, MAPK, and hypoxia-inducible factor-1α (HIF-1α). Newly published studies demonstrate that elevated mtROS contribute to the maintenance of glioma stem cells (GSCs), which are highly tumorigenic and responsible for tumor recurrence and resistance to conventional therapies [48,58].

Mitochondrial dysfunction in GB has been established as a key factor in the development of the Warburg effect, in which tumor cells preferentially metabolize glucose via aerobic glycolysis despite sufficient oxygen [59]. Although this chemical pathway generates less ATP per glucose molecule than oxidative phosphorylation, it can activate the rapid production of metabolic intermediates required for most basic physiological processes, such as nucleotide, amino acid, and lipid biosynthesis. In addition, glycolysis can produce ATP more rapidly than mitochondrial respiration, allowing tumor cells to adapt to the hypoxic microenvironment characteristic of glioblastoma [59,60].

Several studies have demonstrated that the metabolic phenotype of glioblastoma is considerably more complex than originally proposed by Warburg. In a study by Duraj et al., it was found that GSCs and progenitor cells are less glycolytic than differentiated glioma cells. GSCs do not rely solely on aerobic glycolysis; instead, they regulate their metabolism between glycolysis and oxidative phosphorylation, thereby maintaining self-renewal, survival, and therapeutic resistance [61]. GSCs are located in the perivascular niche, which has relatively high levels of oxygen and nutrients, and they prefer to use glucose as the carbon source while maintaining mitochondrial activity. Pyruvate kinase M1 (PKM1) expression increases pyruvate flux into the mitochondria, thereby enabling efficient ATP production via the citric acid cycle and oxidative phosphorylation [62]. Consequently, stem cells consume less glucose, produce less lactate, and maintain higher ATP levels than their differentiated progeny, correlating with a higher mitochondrial reserve capacity and suggesting that GSCs could also depend on oxidative phosphorylation for their progression [61,63]. Metabolic pathways increasingly shift flexibly from glycolysis to oxidative phosphorylation in glial cells, which do not rely on glycolysis alone. This switching is linked to oxygen and nutrient availability, as well as interactions with the tumor microenvironment [64,65]. In particular, glioma stem cells rely on oxidative phosphorylation, and fast-growing tumor cells exhibit increased glycolytic metabolism. This heterogeneity plays a major role in the development of therapeutic resistance and tumor recurrence, making mitochondrial metabolism a promising therapeutic target [66,67].

Another crucial aspect of the Warburg effect is its impact on cellular redox homeostasis. An increase in glucose uptake and glycolytic flux provides a greater supply of metabolic intermediates to support the pentose phosphate pathway, which produces NADPH needed to regenerate antioxidants [68]. Simultaneously, persistent mitochondrial dysfunction continues to generate ROS, creating a paradoxical state in which glioblastoma cells maintain elevated ROS levels while enhancing their antioxidant capacity to prevent oxidative damage. Moderate ROS concentrations promote DNA damage, genomic instability, epigenetic alterations, and the activation of redox-sensitive transcription factors such as HIF-1α, NF-κB, and NRF2, all of which facilitate tumor progression, angiogenesis, invasion, and resistance to radiotherapy and chemotherapy [31,69]. Thus, mitochondrial dysfunction and the Warburg effect cooperate to establish a metabolically adaptive and redox-balanced tumor environment that supports glioblastoma development and progression while providing multiple opportunities for therapeutic intervention [59,60].

The growing understanding of mitochondrial dysfunction and metabolic reprogramming in glioblastoma has spurred the development of therapies aimed at disrupting tumor energy supplies and redox homeostasis [70]. Given the importance of this pathway in tumor aggressiveness, the inhibition of pyruvate dehydrogenase kinase (PDK) using dichloroacetate (DCA) has been investigated because DCA promotes pyruvate dehydrogenase activity, thereby redirecting pyruvate from aerobic glycolysis toward mitochondrial oxidative phosphorylation [70,71].

This metabolic change partially rescues the Warburg phenotype, increases mitochondrial ROS production, induces apoptosis, and sensitizes tumors to radiotherapy and chemotherapy [70]. DCA was shown to penetrate the blood–brain barrier in early-phase clinical trials in glioblastoma patients and was generally well tolerated, with some patients demonstrating prolonged disease stabilization and potential metabolic modulation. Yet its clinical effectiveness is still uncertain, and larger randomized trials are needed to establish therapeutic efficacy [72].

Inhibition of oxidative phosphorylation has also been identified as a way to target mitochondrial metabolism. Metformin, a drug commonly prescribed for the treatment of type 2 diabetes, inhibits mitochondrial Complex I, depletes ATP, activates AMP-activated protein kinase, and suppresses mammalian target of rapamycin (mTOR) signaling. Several Phase I and Phase II clinical trials have studied the use of metformin in combination with standard radiotherapy and TMZ in newly diagnosed glioblastoma [73]. Although these studies confirmed that the combination is generally safe and biologically reasonable, improvements in overall survival have been modest, suggesting that metabolic heterogeneity and adaptive resistance mechanisms limit the efficacy of Complex I inhibition alone.

One of the most important strategies is to inhibit glutamine metabolism, which supplies carbon and nitrogen to the citric acid cycle and to antioxidant synthesis in glioblastoma cells [74]. Telaglenastat, a selective glutaminase inhibitor, is currently in early-phase clinical trials in patients with advanced solid tumors, including high-grade gliomas, as a single agent or in combination with radiotherapy and other targeted agents. Preclinical studies have demonstrated that glutaminase inhibition decreases glutathione synthesis, disrupts redox homeostasis, increases intracellular ROS accumulation, and sensitizes glioblastoma cells to radiation-induced oxidative damage. Although clinical data remain preliminary, these findings support glutamine metabolism as a promising therapeutic target [74].

In addition to bioenergetics, mitochondrial metabolism acts as an epigenetic thermostat, connecting metabolism to chromatin remodeling and impacting tumor evolution and cellular plasticity. However, the activity of histone- and DNA-modifying enzymes is also regulated by mitochondrial metabolites, such as acetyl-CoA, α-KG, succinate, and NAD+, which dynamically modulate gene expression under metabolic stress [75]. These metabolic circuits enable epigenetic hardening of adaptive transcriptional programs that drive tumor persistence and evolution. This metabolic–epigenetic axis is also involved in therapy-induced senescence (TIS), in which mitochondrial dysfunction triggers retrograde signaling that remodels the epigenetic landscape, thereby reinforcing senescence-associated phenotypes [76]. Accordingly, the use of TIS should not be interpreted as a result of cytotoxic treatment but rather as a metabolically and epigenetically programmed state of the cell, making mitochondrial metabolism an interesting target to prevent tumor adaptation and recurrence [76].

- Citric acid cycle enzymes and ROS production

The main factor related to glioblastoma identification is isocitrate dehydrogenase (IDH1/IDH2), which normally converts isocitrate to α-ketoglutarate (α-KG) and produces NADPH that is crucial for the regeneration of reduced glutathione (GSH) and the regulation of ROS [77,78]. IDH1 activity increases the NADPH/NADP+ ratio in the cytoplasm, stimulating lipid synthesis and boosting the cell’s resistance to oxidative stress. This is why even wild-type IDH1 is being considered a target in primary GB: genetic inhibition of IDH1 leads to diminished lipid and deoxynucleotide biosynthesis and increased ROS production, due to decreased α-KG and NADPH, making cells more susceptible to radiation- and erlotinib-induced apoptosis [77].

The association between α-KG and pyruvate dehydrogenase is implicated in promoting of oxidative decarboxylation of some elements, which leads to the production of higher levels of ROS than Complex I [41]. Furthermore, α-KGDH is highly sensitive to elevated ROS levels, which contribute to its damage, impairing its function and potentially disrupting the Krebs cycle. This can lead to a further increase in ROS generation [79].

- 8-hydroxy-2-deoxyguanosine/OGG1 and ROS production

Hydrogen peroxide is formed as a result of the dismutation of superoxide anions or is generated from physiological synthesis in the peroxisome. The carcinogenesis phenotype is associated with cellular damage. However, the hydroxyl radical is more harmful due to its unstable electron configuration, which increases its reactivity with other molecules. It is mainly involved in DNA damage, generating 8-hydroxy-2-deoxyguanosine (8-OHdG), which results from the hydrolysis of 8-hydroxiguanosine (8-OHG) and leads to the mispairing of guanine with adenine [34,36].

8-Hydroxy-2-deoxyguanosine is involved in creating errors during DNA replication, leading to mutations. It is also investigated for its role in the development of genomic instability and other epigenetic modifications [80,81]. An estimated 20,000 nucleobases in DNA are damaged due to the elevated oxidative potential created by this premutagenic product [82]. Thus, 8-OHdG could serve as a crucial marker of oxidative stress that disrupts cellular homeostasis.

On the other hand, 8-oxoguanine DNA glycosylase 1 (OGG1) is a key DNA glycosylase that repairs 8-OHdG. Dysfunction or dysregulation of OGG1 has significant implications for cancer initiation, progression, and therapy resistance, given its role as a protective phenotype against the damaging effects of oxidative stress [83]. OGG1 recognizes and excises 8-OHdG via the base excision repair pathway, preventing the transversion of G/C to T/A. Mutations in the gene encoding this protein are highly associated with cancer promotion and cell proliferation. Several mechanisms could be affected by OGG1 alterations, such as downregulation/loss of function, which leads to increased levels of oxidative DNA damage; mutations (e.g., the Ser326Cys polymorphism) reduce its repair activity; overexpression promotes tumor cell survival by repairing oxidative DNA damage; and epigenetic silencing (promoter methylation) leads to reduced OGG1 expression, thereby altering its main role and promoting survival under oxidative stress conditions [83,84].

Lian et al. examined 45 human glioma tumors and found elevated levels of 8-OHdG in glioma tissues. They also found that high-grade tumors expressed higher levels of LC3B, a protein involved in autophagy, and that there was a correlation between 8-OHdG and LC3B expression. Autophagy inhibition with 3-methyladenine (3-MA) led to an accumulation of ROS in U87 glioma cells. The authors thus suggested that autophagy might play a protective role in glioma cell survival under extreme oxidative stress [85]. Increased oxidative stress has also been associated with oncogenic signaling in glioblastoma, as evidenced by a correlation between epidermal growth factor receptor (EGFR) overexpression and 8-hydroxyguanosine staining in tumor tissue [86]. This is especially important, as oxidized guanine lesions are highly mutagenic and can cause genomic instability if not repaired efficiently [80]. In addition to its canonical DNA repair role, OGG1 may also be involved in other transcription-related and cellular signaling tasks, suggesting that the OGG1/8-oxoguanine axis could have regulatory roles in cancer cells [81].

Pharmacological inhibition of OGG1 has been identified as a strategy to disrupt the repair of oxidative DNA damage in glioblastoma. Studies on the inhibitory effects of the OGG1 inhibitor TH5487 showed that it is able to inhibit the growth of a variety of cancer cell lines, including glioblastoma, and mechanistic studies revealed that OGG1 inhibition impairs the repair of 8-oxoguanine (8-oxoG) and results in the accumulation of 8-oxoG in the genomic DNA of cells [87,88]. Moreover, the inhibition of OGG1 during oxidative stress is associated with the accumulation of oxidized DNA bases, telomere instability, and reduced cell proliferation. OGG1-mediated repair is an important process for cellular tolerance to oxidative stress [89,90]. All of these results suggest that the level of oxidative DNA damage (measured by 8-oxoG) and the base-excision repair pathway mediated by OGG1 could play a role in glioblastoma cells’ capacity to resist increased oxidative and genotoxic stress. Nevertheless, there was a very limited direct connection between the activity of OGG1, the accumulation of 8-oxoG, and the therapeutic response in glioblastoma, which needs to be further investigated [87,89].

- NADPH family and ROS production

Nicotinamide adenine dinucleotide phosphate oxidases (NADPH oxidases) are a family of enzymes that represent the main source of ROS. NADPH oxidases and mitochondria work together to regulate ROS levels in response to a variety of stimuli, such as growth factors and cytokines [91]. The NOX family, or NADPH oxidases, consists of seven isoforms: NOX1 to NOX5, DUOX1, and DUOX2. They are expressed in phagocytes and immune cells and are known to have a phagocytic function. However, NOX1, NOX4, and NOX5 are highly implicated in elevated ROS production, redox signaling, calcium-dependent ROS generation, and H_2_O_2_ production [92,93]. NOX enzymes catalyze the transfer of electrons from NADPH to oxygen, forming superoxide and hydrogen peroxide, and contribute to redox imbalance. Excess ROS produced by NOX enzymes (especially NOX1, NOX4, and NOX5) induces 8-OHdG lesions, leading to mutations and genomic instability. NOX-driven oxidative stress promotes p53 mutations and activates anti-apoptotic proteins (Bcl-2, Bcl-xL) [94,95].

NOX2 and NOX4 contribute to chronic inflammation by activating NF-κB and inducing pro-inflammatory cytokines (IL-6, TNF-α), which fuel the pro-tumorigenic microenvironment and enhance the activation of redox-sensitive pathways such as PI3K/Akt, MAPK, and NF-κB [93].

NOX2, also known as cytochrome b-245 beta chain (CYBB), is a membrane-bound enzyme complex originally identified in immune system cells, endothelial cells, and smooth muscle cells. It is implicated in the generation of superoxide upon activation. NOX2 catalyzes the transfer of electrons from NADPH to oxygen, generating superoxide anions, which can be converted into hydrogen peroxide, hydroxyl radicals, and peroxynitrite. Through its activity, NOX2 suppresses anti-tumor immunity by inhibiting T cells (CD8+ cytotoxic T cells), thereby reducing immune surveillance [96,97].

NADPH Oxidase 4 (NOX4) is a member of the NOX family of enzymes. Unlike other NOX family members, it is primarily known for its constitutive generation of reactive oxygen species, particularly hydrogen peroxide [92], which is involved in various physiological and pathological processes and influences numerous cellular processes critical to cancer development and progression [98,99].

While ROS are essential for normal cellular functions, excessive or dysregulated ROS production by NOX4 can contribute to oxidative stress, leading to cellular damage (DNA, proteins, lipids), and can play a dualistic role in cancer, either inducing cytotoxicity or regulating pro-survival pathways. NOX4 is primarily described as a factor in tumorigenesis, promoting cell proliferation and survival. For instance, NOX4-derived ROS can activate signaling pathways that promote cancer cell proliferation and survival. In renal cell carcinoma (RCC), NOX4 supports tumorigenesis by promoting the expression and nuclear accumulation of hypoxia-inducible factor 2-alpha (HIF2α), a transcription factor that drives the expression of genes involved in angiogenesis and metabolism; NOX4 has been shown to promote tumor cell proliferation and growth in many cancers, including non-small cell lung cancer (NSCLC), thyroid cancer, and ovarian cancer [100,101].

NOX4 appears to be particularly relevant in glioblastoma, where it has been reported to be elevated in tumor tissues and glioblastoma stem cells. New findings suggest that NOX4-dependent ROS are involved in multiple processes that contribute to glioblastoma aggressiveness, including proliferation, self-renewal, invasion, angiogenesis, and adaptation to hypoxia [102].

In patient-derived glioblastoma stem cells, NOX4 was identified as a downstream effector of TGF-β signaling. It was more highly expressed in stem-like cells than in differentiated tumor cells, suggesting a potential role for NOX4-dependent ROS signaling in maintaining the glioblastoma stem-cell phenotype [103]. Importantly, recent studies have shown that NADPH oxidase activity is also required for glioblastoma cells’ response to hypoxia and radiotherapy [104]. Overall, these results indicate that NOX-induced ROS are not just a by-product of the oxidative stress occurring in glioblastoma but could possibly act as signaling mediators enabling the survival and metabolic adaptation of tumor cells to therapeutic stress. Thus, the NOX–ROS axis, particularly NOX4-mediated signaling, could be a novel approach to blocking redox adaptation and enhancing the drug susceptibility of GB cells [92,103].

- Glutathione peroxidases (GPxs)

Glutathione peroxidases (GPxs) are a family of selenium-dependent antioxidant enzymes that reduce hydrogen peroxide (H_2_O_2_) and organic hydroperoxides using glutathione (GSH) as a substrate. Their main role is to prevent oxidative stress by maintaining redox homeostasis in cells. These proteins are present in the body in several isoforms, including GPx1 (ubiquitously expressed in the cytoplasm and mitochondria), GPx2 (mainly expressed in the gastrointestinal tract), GPx3 (a secreted form found in plasma), and GPx4 (which reduces lipid hydroperoxides and plays a role in ferroptosis regulation) [105] (Table 2).

The glutathione peroxidase enzymes have been implicated in cancer, acting as both tumor suppressors and tumor promoters, depending on the cell type, tumor stage, and oxidative environment.

With further investigation into their tumor suppressor roles, GPx enzymes have been associated with antioxidant defense mechanisms by detoxifying ROS (e.g., H_2_O_2_), anti-inflammatory effects by limiting oxidative stress generated by ROS, and indirect suppression of chronic inflammation, a key driver in many cancers (e.g., colorectal, gastric) [37]. Moreover, epigenetic modifications of GPx3, such as promoter hypermethylation, have been associated with several cancers (colorectal cancer, gastric cancer and breast cancer) [106,107].

On the other hand, these biomarkers could play tumor-promoting roles, notably by protecting cancer cells by blocking ROS-induced apoptosis, leading to overexpression of GPx1 or GPx4, which could induce resistance to chemotherapy (e.g., cisplatin, doxorubicin), allowing these cells to survive under oxidative stress (Table 2) [108].

Glioblastoma’s antioxidant defense mechanism plays a crucial role in combating reactive oxygen species and mitigating oxidative stress, and glutathione peroxidases are a key part of this mechanism. Within the GPX family, GPx1 has been identified as a key factor in maintaining hydrogen peroxide homeostasis in glioblastoma, especially under hypoxic conditions [109]. Recent studies have shown that hypoxia stimulates glioblastoma cells to produce more H_2_O_2_ and induces hypoxia-inducible factor-1α (HIF-1α)-dependent transcription of GPx1 [109]. GPx1, in turn, plays a role in detoxifying excess hydrogen superoxide, reducing oxidative stress and thus preventing glioblastoma cells from undergoing apoptosis [109]. On the other hand, GPx1 depletion led to hydrogen superoxide accumulation, increased apoptotic cell death, and reduced tumor growth in glioblastoma xenograft models [109]. Interestingly, hypoxia also stimulated the release of GPx1 from EVs, thereby preventing hydrogen superoxide- or radiation-induced apoptosis in glioblastoma and endothelial cells, suggesting that extracellular GPx1 may regulate the oxidative-stress response in the tumor microenvironment [109]. These results are in line with previous studies demonstrating that variations in GPx1 expression influence the sensitivity of glioblastoma cells to oxidative stress, and that these differences in GPx1 expression account for the heterogeneity in the capacity of GB cells to endure ROS/RNS-induced damage [110]. In summary, the combined evidence indicates that regulating hydrogen superoxide levels via GPx1 could be a crucial process by which glioblastoma cells cope with oxidative and hypoxic stress and may contribute to their therapeutic resistance (Table 2) [109,110].

**Table 2:** Functional and mechanistic roles of GPx isoforms in cancer biology.

GPx	Role in Cancer	Mechanism	Clinical Relevance	References
GPx1	Tumor suppressor/promoter	Controls H_2_O_2_; protects DNA	Overexpression → chemoresistance	[110,111,112]
GPx2	Context-dependent	Gut homeostasis; Wnt signaling	Overexpressed in colorectal cancer	[113,114]
GPx3	Tumor suppressor	Silenced via promoter methylation	Biomarker & epigenetic target	[106]
GPx4	Tumor promoter	Prevents lipid peroxidation/ferroptosis	Target for inducing ferroptosis	[112,115]

- Superoxide dismutase

The superoxide dismutase family (SOD), especially its main isoforms SOD1, SOD2, and SOD3, has recently been the subject of extensive investigation. Their connections with cancer progression, development, and therapy are mainly highlighted by their classical antioxidant role and newly discovered regulatory and pathological roles [116].

SOD enzymes are essential antioxidant enzymes that catalyze the dismutation of the superoxide radical into less reactive hydrogen peroxide and oxygen [49]. There are mainly three isoforms with distinct cellular locations. SOD1 is ubiquitously expressed in various cellular compartments, primarily in the cytosol, nucleus, and mitochondrial intermembrane space [117]. SOD2 is predominantly located in the mitochondrial matrix, and SOD3 is maintained in the extracellular matrix and cell membrane [118,119]. The role of these enzymes extends beyond detoxification; the SOD family participates in redox signaling as a second messenger, influencing pathways such as growth, metabolism, and cell survival [120]. For instance, SOD1 can translocate to the nucleus, bind to promoters, and regulate the expression of genes involved in the oxidative stress response and DNA repair [118].

The implication of the SOD family in cancer progression is largely due to elevated ROS levels in cancerous cells, which lead to DNA damage and genomic instability. To resist these high-ROS conditions, cancer cells often rely on robust antioxidant defenses, including SODs, to neutralize excess ROS and avoid ROS-induced apoptosis [49,121]. These enzymes thereby play an important role in protecting cells from ROS-mediated damage and may suppress early tumorigenesis (Table 3). However, once the cell is transformed, this metabolism is hijacked to promote the survival and growth of cancerous cells [119].

The major mitochondrial SOD type, SOD2 (MnSOD), is thought to play a significant role in glioblastoma therapeutic resistance and redox homeostasis [120]. Increased expression of SOD2 has been linked to glioblastoma tumor-initiating cell populations and TMZ resistance, and inhibition of SOD2 has been shown to increase temozolomide-induced oxidative stress and re-sensitization to TMZ in experimental models [121,122]. More recent studies highlight the role of mitochondrial ROS regulation in GB survival and response to therapeutic interventions [123]. Taken together, these results indicate that the regulation of superoxide by SOD may be involved in glioblastoma cells’ adaptation to oxidative stress and could represent a vulnerability for therapeutic intervention (Table 3) [122,123].

#### 4.1.2. Biomarkers Related to Peroxisome Oxidative Metabolism

Peroxisomes are essential cellular organelles; their primary role is to generate ROS via oxidases. These enzymes use oxygen as their primary substrate to catalyze various biochemical reactions, producing ROS as byproducts [124]. During the enzymatic reaction, peroxisomes can cause some electrons to leak from the electron transport chain; these electrons are highly reactive with oxygen, leading to the formation of superoxide radicals, which are subsequently converted to hydrogen peroxide [124]. It is important to note that peroxisomes have a robust antioxidant system to control ROS levels. This system includes the enzyme catalase, which catalyzes the breakdown of hydrogen peroxide into water and oxygen. This balance between ROS production and scavenging is crucial for maintaining cellular homeostasis [124,125].

- Peroxisome proliferator-activated receptor (PPARα)

Peroxisome proliferator-activated receptor alpha (PPARα) is one of the three major isoforms of nuclear receptors in the PPAR family (PPARα, PPARβ/δ, PPARγ) [126]. PPARα is a ligand-activated transcription factor and a member of the nuclear receptor family. It primarily regulates genes involved in fatty acid transport and β-oxidation, as well as energy homeostasis and inflammation [125]. This biomarker is mainly expressed in tissues with high rates of fatty-acid oxidation (FAO), such as the liver, heart, muscle, and kidney; besides metabolism, PPARα is also implicated in major cellular pathways such as proliferation, differentiation, and oxidative stress, since it can act as a sensor and regulator of cellular energy, thereby affecting ROS production (Table 3) [127].

In various cancer models, such as melanoma, lung carcinoma, glioblastoma, and fibrosarcoma, PPARα agonists suppress proliferation, often by inhibiting endothelial cells, and reduce pro-angiogenic lipid mediators [128]. Based on multiple reports, the activation of this marker can reprogram the cell by promoting fatty acid oxidation and reducing glucose uptake, thereby impairing “Warburg-like” metabolism [129,130]. For instance, in certain cancer cell lines, PPARα activation reduced the expression of glucose transporters and glycolytic enzymes, decreased ATP production from glycolysis, and increased oxidative stress, thereby enhancing proliferation [131].

Peroxisome proliferator-activated receptors (PPARs) are ligand-dependent transcription factors that regulate lipid metabolism, cellular differentiation and redox homeostasis, among which PPARγ is the best-studied receptor in glioblastoma [127]. PPARγ is especially highly expressed in mesenchymal glioblastoma stem cells, and activation of PPARγ has been demonstrated to reduce tumor growth and stemness by blocking STAT3 signaling pathways and the proneural-to-mesenchymal transition [132]. PPARγ activation could also modulate redox status in glioma cells by increasing ROS levels, leading to oxidative stress and glioma cell death. Furthermore, PPARα and PPARγ control a number of lipid-metabolic pathways, which are identified as significant contributors to glioblastoma aggressiveness and therapeutic resistance [133]. Overall, these results indicate that the activation of PPAR might link metabolic and redox pathways in glioblastoma, but the impact of PPAR activation seems to be context- and subtype-specific and needs further study [134,135].

- Catalase

Catalase is an enzyme primarily located in and functioning within peroxisomes; its primary function is to regulate cellular redox balance by decomposing hydrogen peroxide into water and oxygen molecules [136]. Under homeostatic conditions, catalase is a tumor suppressor; however, in some highly aggressive tumors, antioxidant defenses are disrupted by catalase upregulation, and cancer cells adapt to the high ROS environment. In this context, it protects the cancer cell and can promote proliferation, survival signaling, metabolic reprogramming, immune evasion, or angiogenesis [137,138]. Elevated catalase production could even be the main cause of therapeutic resistance, since it detoxifies hydrogen superoxide produced by chemotherapy or radiotherapy agents that lead cancer cells to undergo apoptosis [137]. Recently, some cancer therapy strategies have not aimed to increase antioxidant capacity, but rather to inhibit catalase (or other antioxidant defenses), thereby overwhelming cancer cells with ROS and triggering cell death [137,139].

Intracellular levels of hydrogen superoxide are lower in the presence of CAT in glioblastoma, and this is associated with increased TMZ and radiotherapy resistance [139]. In terms of mechanism, removal of hydrogen superoxide by CAT protects glioblastoma cells from oxidative stress, while blocking CAT results in enhanced hydrogen superoxide accumulation and decreased proliferation of tumor cells (Table 3) [139]. Moreover, CEBPD has been shown to influence CAT expression and hydrogen superoxide clearance, thereby linking antioxidant defense with glioblastoma survival via CAT [140]. All of this suggests that increased CAT activity could enable glioblastoma cells to cope with oxidative stress and avoid ROS-mediated therapeutic damage.

### 4.2. Exogenous Generation of ROS

#### 4.2.1. Ionizing Radiation (IR)

**Figure 1:**
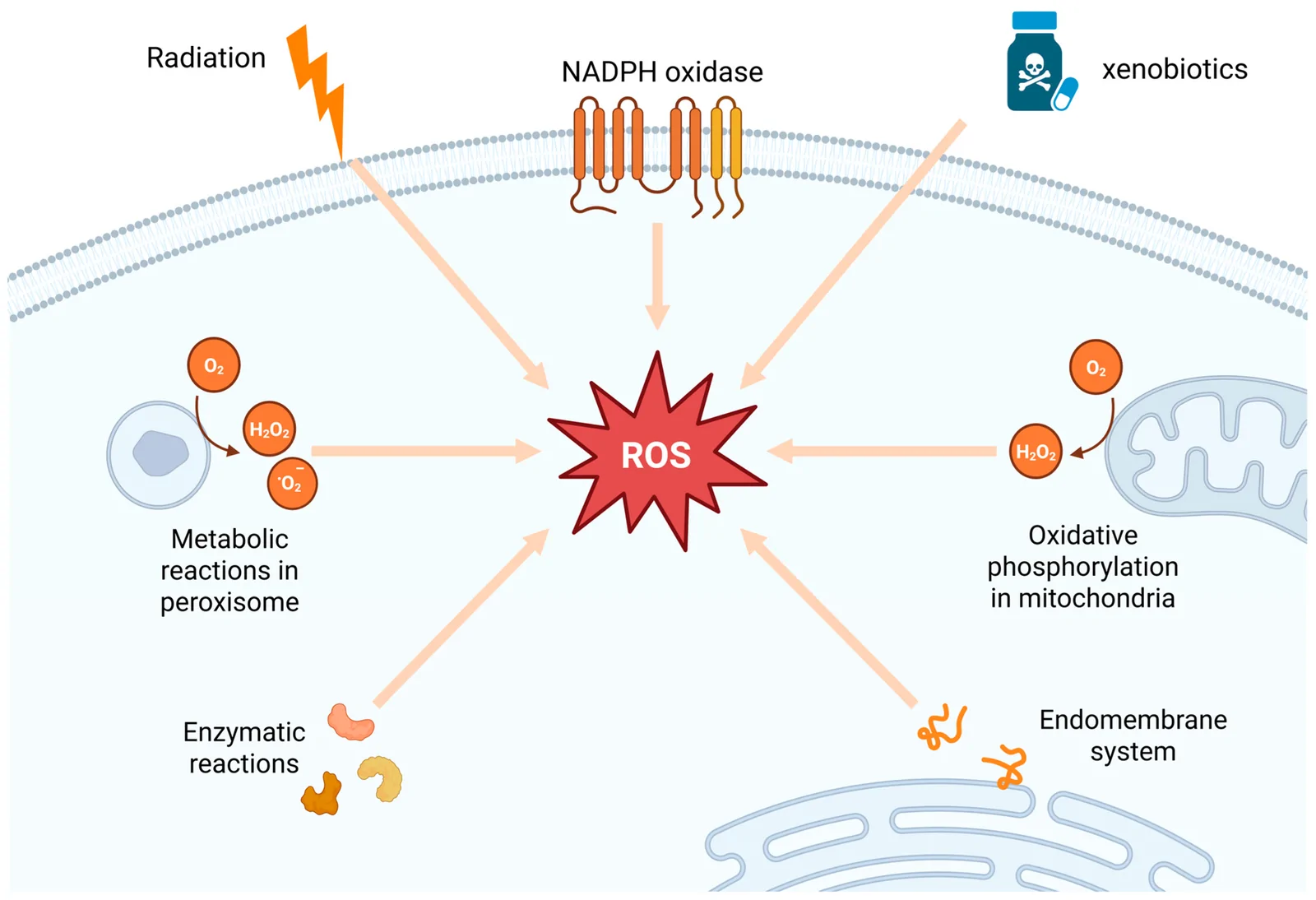
Cellular network of the generation of reactive oxygen species. Reactive oxygen species (ROS) are generated from several cellular processes, including mitochondrial respiration, peroxisomal metabolism, enzymatic reactions, and NADPH oxidase activity. External factors such as radiation and xenobiotics can further stimulate ROS production. These sources collectively contribute to the intracellular ROS pool. Created in BioRender. Lamrabet, S. (2026) https://BioRender.com/uy4vkms (accessed on 1 September 2026).

Ionizing radiation (IR) is the main component of radiotherapy, generating a massive amount of ROS in cancer cells, which accounts for approximately 60–70% of radiation-induced biological damage through two main mechanisms: indirect action (radiolysis of water) and direct action (targeting biomolecules) [141]. These forms of radiation typically include X-rays, gamma rays, and charged particles that interact with cells by ionizing water molecules, thereby generating ROS at an accelerated rate compared to that in normal tissues [44,142]. Beyond the main ROS production channel, IR promotes excessive mitochondrial activity by boosting several pathways, such as cell respiration (leading to persistent dysfunction of the electron transport chain) and membrane potential, which are highly involved in the generation of oxidative species [143,144] (Figure 1). It is also implicated in causing damage to DNA double strands, resulting in cell cycle arrest and ferroptosis [141,145].

Ionizing radiation induces cell cycle arrest, triggering the accumulation of oxidative radicals and stimulating the NADPH oxidase family [146]. Furthermore, persistent or excessive ROS production also mediates radiation-induced normal tissue injury (Figure 1). Oxidative stress activates pro-inflammatory and pro-fibrotic pathways, contributing to acute toxicities such as mucositis and dermatitis, as well as late complications including pulmonary fibrosis, cardiovascular damage, neurotoxicity, and gastrointestinal injury [141].

#### 4.2.2. Xenobiotics

Xenobiotics are chemical compounds foreign to biological organisms; they include cigarette smoke constituents and chemical pollutants (e.g., benzene, polycyclic aromatic hydrocarbons (PAHs), heavy metals) [147]. These compounds are mainly metabolized in cells, often via cytochrome P450 and NADPH reductase, leading to a massive production of reactive oxygen species (Figure 1). These components predominantly produce additional oxygen molecules to detoxify the toxins accumulated in cancer cells. However, uncoupling mechanisms arising from binding failure could primarily drive electron leakage through the electron transport chain, directly forming the superoxide radical [147,148].

Quinones are organic compounds with a cyclic dione structure found in some dyes, pollutants like PAHs, and drugs like doxorubicin. These chemicals are reduced by the activation of NADPH reductase and cytochrome P450, generating unstable radicals that bind strongly to oxygen molecules, thereby forming a continuous loop of ROS production called redox cycling. Some other pollutants, especially heavy metals such as lead, cadmium, and mercury, disable the activation of the antioxidant system (i.e., SOD, catalase, and glutathione) [149].

**Table 3:** Oxidative stress-related biomarkers in cancer: biological significance and role in tumor development [82].

Physiological Process Altered	Biomarker	Biological Significance	Role in Cancer Development
DNA Oxidation Products	▪ 8-Hydroxy-2’-deoxyguanosine (8-OHdG)	Oxidative damage to guanine bases in DNA	Causes mutations, genomic instability, and cancer initiation
	▪ 8-Oxoguanine	Enzyme that repairs 8-OHdG lesions [82]	Reduced activity leads to the accumulation of mutations in cancer cells
Enzymatic Antioxidants	▪ Superoxide dismutase (SOD)	Converts superoxide anion (O_2_•−) into hydrogen peroxide (H_2_O_2_) [49,117]	Altered expression affects the tumor microenvironment; high levels in some cancers, such as glioblastoma, breast cancer, colorectal cancer, and lung cancer (NSCL) [150,151].
	▪ Catalase (CAT)	Breaks down hydrogen peroxide into water and oxygen [136,139]	Decreased levels in tumors allow for ROS accumulation and DNA damage
	▪ Glutathione peroxidase (GPx)	Detoxifies peroxides to prevent oxidative damage	Elevated in some cancers (colorectal cancer, pancreatic ductal adenocarcinoma, and ovarian cancer), contributing to therapy resistance [151,152].
Non-Enzymatic Antioxidants	▪ Reduced glutathione levels	Maintains redox homeostasis	Increased levels in tumor cells enhance drug resistance
	▪ Glutathione (GSH/GSSG)	Indicator of oxidative stress	A high GSSG/GSH ratio suggests a redox imbalance in cancer
	▪ Vitamin C & E	Neutralizes free radicals	Deficiency increases oxidative stress and cancer risk
Reactive Oxygen Species (ROS) & Reactive Nitrogen Species (RNS)	▪ Superoxide anion (O_2_•−)	Generated by mitochondria and NADPH oxidases	Promotes cancer cell survival and proliferation at moderate levels; at high levels, it induces apoptosis
	▪ Hydrogen peroxide (H_2_O_2_)	Byproduct of cellular metabolism and oxidase activity	Involved in redox signaling, angiogenesis, and metastasis
Oxidative Stress-Related Genes & Transcription Factors	▪ NRF2	Master regulator of antioxidant defense	Overexpression in cancer enhances chemoresistance
	▪ KEAP1	NRF2 inhibitor that regulates oxidative stress response	KEAP1 mutations lead to persistent NRF2 activation in tumors
	▪ p53	Regulates oxidative stress-induced apoptosis	Mutations cause loss of oxidative stress control, promoting cancer progression
	▪ NF-κB	Regulates inflammation and cell survival	Persistent activation promotes chronic inflammation and tumorigenesis
	▪ HIF-1α	Regulates cellular response to hypoxia and oxidative stress	Induces angiogenesis and metabolic reprogramming in tumors
Lipid Peroxidation Products	▪ Malondialdehyde (MDA)	End product of polyunsaturated fatty acid peroxidation	Elevated levels correlate with tumor growth, metastasis, and poor prognosis
	▪ 4-Hydroxynonenal (4-HNE)	Reactive aldehyde that modifies proteins and DNA	Induces mutations, promotes cancer cell proliferation, and modulates signaling pathways
	▪ F2-Isoprostanes	Stable lipid peroxidation marker	Associated with chronic inflammation and tumor progression

## 5. Discussion

### 5.1. The Interplay Between Glioblastoma Heterogeneity and the Tumor Microenvironment

The high molecular and metabolic heterogeneity of GB may be an important determinant of how tumor cells respond to oxidative stress and therapeutic pressure. Instead of being a homogeneous disease, GB is an example of a genetically and phenotypically heterogeneous population of cells that may undergo different kinds of redox adaptation to endogenous or therapeutic stress sources. The distinct genetic and epigenetic changes, such as *EGFR* amplification, *TP53* and *PTEN* mutations, *TERT* promoter mutations, and *IDH* mutation status, indicate that redox regulation and metabolic dependencies may not be uniform across GB tumors [153]. Single-cell RNA-seq of GB tumor cells showed that they are in a variety of dynamic states: astrocyte-like, oligodendrocyte progenitor-like, neural progenitor-like, and mesenchymal-like; that these states are present in the same tumor cells; and that they shift over time, depending on genetic changes and the microenvironment [153]. This remarkable plasticity may contribute to resistance to metabolic and oxidative stress, tumor recurrence, and therapeutic resistance. Besides intra-tumor cell diversity, GB is also embedded in a very complex microenvironment featuring glioma-associated microglia/macrophages, astrocytes, endothelial cells, pericytes, fibroblast-like stromal cells, lymphocytes, components of the extracellular matrix, and hypoxic niches [154,155].

There is increasing evidence that tumor-associated macrophages are not simply present in the GB microenvironment, but actively participate in its immunosuppressive properties, as evidenced by the fact that they acquire a dominant immunosuppressive phenotype characterized by the production of anti-inflammatory cytokines such as TGF-β and IL-10 and by their function in promoting angiogenesis, invasion, and extracellular matrix remodeling [156,157]. The question arises as to how hypoxia-induced signaling relates to this immunosuppressive program: stabilization of HIF-1α under hypoxia seems to promote VEGF-mediated angiogenesis, a metabolic shift towards glycolysis, maintenance of stemness, and production of more ROS; these mechanisms could be combined into a self-reinforcing loop that sustains tumor progression and resistance to radio-chemotherapy [158,159]. In this view, the interaction between the genetically heterogeneous GB cells and the tumor microenvironment (TME) can be regarded as an ecosystem that might influence the evolution of GB, diminish immunity, trigger metabolic adaptation, and promote treatment resistance. This interpretation reinforces a general principle in the field: Future treatment strategies might require addressing the TME concurrently with molecular changes in GB, rather than treating them as independent issues [156].

All these observations indicate that the relationship between glioblastoma cells and their microenvironment is highly dynamic and involves a continuous interplay among hypoxia, increased ROS production, and metabolic adaptations to the persistent therapeutic challenge [160]. These conditions would thus promote redox imbalance and stimulate compensatory antioxidant pathways that would support tumor survival and resistance to treatment [161]. Key regulators of redox homeostasis, such as NOX4 and the KEAP1–NRF2 signaling pathway, form the basis of their investigation as potential drivers and targets of glioblastoma progression and treatment response [162,163].

### 5.2. KEAP1–NRF2 Signaling Axis in Glioblastoma Development and Therapy Resistance

Although glioblastoma cells are intrinsically vulnerable to oxidative stress, they possess highly effective antioxidant defense systems that enable them to survive in conditions of chronic reactive oxygen species buildup. The KEAP1–NRF2 signaling pathway is a crucial regulator of cellular redox balance. Under basal conditions, KEAP1 promotes NRF2 ubiquitination and proteasomal degradation, thereby limiting constitutive antioxidant signaling. Oxidative or electrophilic stress can modify reactive KEAP1 cysteine residues, impairing NRF2 degradation and allowing NRF2 to accumulate and activate antioxidant response element (ARE)-dependent genes [104,164,165]. In glioblastoma, this pathway may therefore function not simply as an antioxidant system, but as an adaptive mechanism that enables tumor cells to tolerate persistent oxidative and therapeutic stress.

NRF2 activation induces a broad cytoprotective program involving antioxidant enzymes, glutathione metabolism, detoxification, mitochondrial protection, and metabolic reprogramming [166,167]. Rather than requiring frequent activating mutations in KEAP1 or NRF2, which appear to be relatively uncommon in glioblastoma, persistent NRF2 activity may arise through dysregulation of upstream oncogenic pathways, including EGFR, PI3K/AKT, mTOR, and HIF-1α, as well as through accumulation of p62/SQSTM1, which can interfere with KEAP1-mediated NRF2 degradation [104,168]. This distinction is important because it suggests that the functional redox phenotype of glioblastoma may be more informative than KEAP1/NRF2 genotype alone when considering NRF2-directed therapeutic strategies.

There is increasing evidence that activation of NRF2 is a component of tumor survival and also a mechanism of metabolic adaptation. NRF2 has been shown to regulate the expression of enzymes involved in the pentose phosphate pathway, glutamine metabolism, and NADPH biosynthesis to maintain a balance of reducing equivalents essential for glutathione regeneration [169,170,171]. This is a paradoxical redox state of glioblastoma cells in which high ROS levels are coupled with strong antioxidant defenses, suggesting the coexistence of elevated ROS production with enhanced antioxidant defenses that limit ROS-mediated cytotoxicity [172,173]. Furthermore, NRF2 has been linked to maintaining mitochondrial function, decreasing mitochondrial ROS, and promoting metabolic switching between glycolysis and mitochondrial respiration in glioma stem cells, as well as mechanisms implicated in tumor recurrence and therapeutic resistance [174].

An important clinical question is whether the KEAP1–NRF2 axis contributes to resistance to radiotherapy and TMZ [175]. NRF2 inhibition was shown to reverse this protective phenotype in glioma cells, supporting a functional link between NRF2 signaling and TMZ resistance rather than a merely correlative association [173]. As a result, NRF2 activation is associated with TMZ resistance mechanisms that involve increased antioxidant defenses and reduced oxidative and ferroptotic damage [176]. TRIM25, an E3 ubiquitin ligase, appears to increase KEAP1 ubiquitination, thereby promoting NRF2 stabilization and nuclear accumulation, which leads to a decrease in oxidative stress, since triggering NRF2-mediated antioxidant defenses could reduce intracellular ROS accumulation and promote tumor cell survival, and thus leads to an increase in TMZ resistance in gliomas [175,177].

However, regarding radioresistance, it is believed that the majority of the cytotoxic effects of ionizing radiation result from the production of ROS, which induces DNA double-strand breaks and oxidative damage [178]. This radiation itself appears to trigger NRF2 signaling simultaneously, leading to the upregulation of antioxidant enzymes, increased synthesis of the antioxidant glutathione, improved DNA repair, and inhibition of apoptosis—thus reducing the efficacy of the therapy itself [179,180].

**Figure 2:**
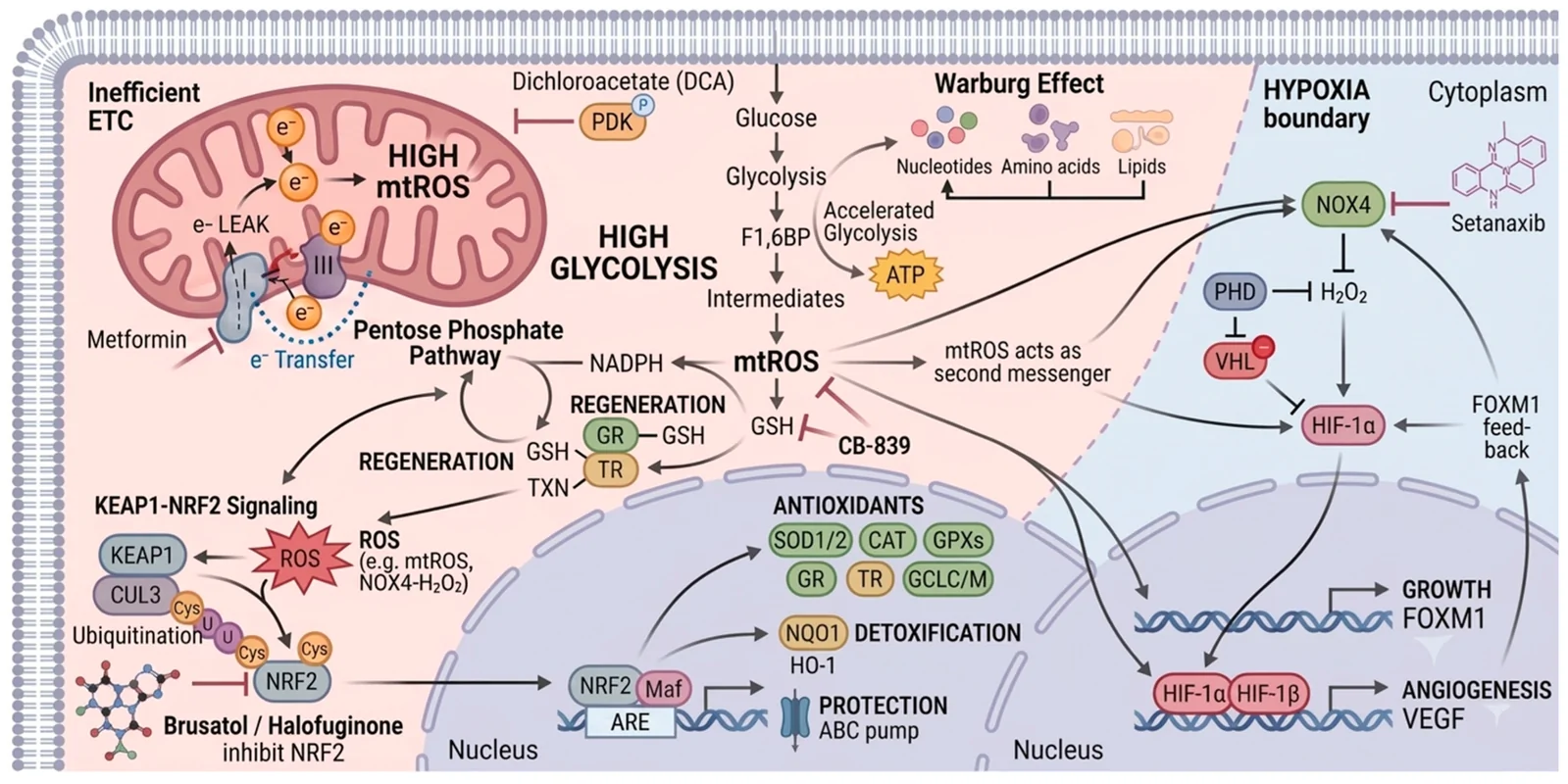
Crosstalk between mitochondrial ROS, metabolic reprogramming, NRF2, and HIF-1α signaling in cancer. Initially created in Created in BioRender. Lamrabet, S. (2026) https://BioRender.com/bd4k0di (accessed on 1 September 2026), further enhanced by Gemini Pro AI.

Based on the preceding discussion, these key findings can be synthesized as follows: NRF2 has become a promising drug target because of its pivotal role in redox regulation [180]. Several preclinical studies, based on cell lines and GSCs, support this rationale, as pharmacological inhibition or genetic silencing of NRF2 results in the accumulation of intracellular ROS, impaired glutathione metabolism, reduced maintenance of glioma stem cells, and a marked improvement in the efficacy of radiotherapy and TMZ [181,182,183]. Preclinical studies using NRF2-targeting approaches, including Brusatol, ML385, and Halofuginone, suggest that NRF2 inhibition can increase oxidative stress and enhance the sensitivity of glioblastoma models to therapy (Figure 2) [183,184]. Similarly, inhibitors of glutathione synthesis, thioredoxin metabolism, and heme oxygenase-1 are under investigation as adjuvants that provide an alternative way to bypass GB cell antioxidant defenses [185,186,187].

However, these findings remain predominantly preclinical, and the therapeutic window for NRF2 inhibition remains an important unresolved issue because NRF2 also protects normal tissues from oxidative injury. Future studies should therefore determine whether NRF2-dependent redox phenotypes can be used to identify patients most likely to benefit from NRF2-targeted therapy and whether selective NRF2 inhibition can be safely combined with TMZ or radiotherapy.

### 5.3. NOX4 and Hypoxia-Induced ROS Generation

The glioblastoma microenvironment is not a static hypoxic environment but one of instability, with areas of chronic hypoxia alongside regions that alternate between cycles of hypoxia and normoxia as a result of disorganized and functionally suboptimal tumor vascularization [188,189]. This difference may not seem significant at first glance, but the data on NOX4 indicate that this is not the case, as GB cells seem to be affected differently by changes in oxygen levels than by anoxia [163,190]. Xenograft studies have revealed that regions of cycling hypoxia, but not chronic hypoxia, have preferentially higher expression of NOX4 and ROS production, and greater radioresistance, while showing a decrease in radioresistance after suppression of NOX4 [163,191]. If this is taken at face value, then NOX4 is more than just a marker for hypoxia; it seems to be actively converting an unstable oxygen environment into a persistent, pro-survival signaling state. This does not clarify whether the observed difference is due to a true difference between hypoxic subtypes or a threshold effect whereby cycling hypoxia leads to a greater production of ROS per unit time than chronic hypoxia, and it is important to remember that ambiguity exists regarding NOX4 as a clean marker of a separate hypoxic subtype before describing it as such [163,190].

In this context, the association of NOX4-derived ROS with HIF-1α is of special significance. Under normoxic conditions, prolyl hydroxylase domain (PHD) enzymes hydroxylate HIF-1α, leading to its recognition by the von Hippel–Lindau (VHL) complex and its ubiquitination and proteasomal degradation. Hypoxia inhibits PHD-dependent hydroxylation and thus stabilizes HIF-1α [192,193]. However, ROS can also have a modulatory effect on this oxygen-sensing mechanism by regulating the activity of redox-sensitive proteins that play a role in the regulation of HIF-1α [194]. Experimental studies have also shown an interaction between NOX4 ROS and HIF-1α in the context of glioblastoma. NOX4-mediated mitochondrial ROS was reported to promote the stabilization of HIF-1α, while NOX4 knockdown, which is associated with diminished NOX activity, decreased the expression of HIF-1α [195]. Therefore, NOX4-mediated ROS can exacerbate the hypoxic response even under conditions of intermittent oxygen changes, allowing for the conversion of transient environmental stress into persistent oncogenic signaling [191,195]. Once stabilized, HIF-1α translocates to the nucleus and heterodimerizes with HIF-1β, activating genes involved in angiogenesis, glycolysis, cell survival, invasion, and adaptation to oxygen deprivation [196]. In GB, this response is particularly advantageous because the tumor must continuously adapt to fluctuating oxygen and nutrient availability. Indeed, cycling hypoxia has been associated with persistent HIF-1α activity and increased radioresistance in GB models, while inhibition of HIF-1α signaling reduces this hypoxia-associated resistance [163,197]. Taken together, these findings suggest that the NOX4–ROS–HIF-1α axis may represent an important molecular mechanism linking the physical characteristics of the GB microenvironment to the activation of transcriptional programs that promote tumor survival and treatment resistance.

An especially important downstream component of this pathway is the transcription factor Forkhead box M1 (FOXM1). FOXM1 is frequently overexpressed in glioblastoma and has been associated with aggressive tumor characteristics and poor clinical outcomes [198,199]. Traditionally, FOXM1 has been recognized as a major regulator of cell-cycle progression, DNA replication, and mitosis. However, increasing evidence suggests that its function extends beyond cell proliferation to include metabolic reprogramming. This is particularly relevant to the NOX4–HIF-1α pathway because recent experimental work has established a direct mechanistic connection between NOX4-derived ROS, HIF-1α, and FOXM1 in GB cells [195,200]. Su et al. (2021) have demonstrated that NOX4-derived mitochondrial ROS induce FOXM1 expression through the stabilization of HIF-1α. Their mechanistic experiments showed that HIF-1α can directly activate FOXM1 transcription, while inhibition of HIF-1α attenuates NOX4-induced FOXM1 expression [195]. Furthermore, NOX4 and FOXM1 were both overexpressed in glioma specimens and were associated with unfavorable clinical outcomes. These findings provide important evidence that FOXM1 may represent a downstream transcriptional effector of NOX4-dependent redox signaling rather than an independent marker of tumor aggressiveness [195,199,201].

This relationship is also consistent with evidence linking NOX4 to other metabolic signaling pathways in GB. NOX4-dependent ROS have been implicated in Akt/HIF-1α-mediated regulation of glycolysis, suggesting that NOX4 may act as a convergence point between oxidative stress, growth-factor signaling, and metabolic adaptation [202,203]. Therefore, the contribution of NOX4 to glioblastoma metabolism may be more complex than a single linear pathway. NOX4-derived ROS may simultaneously influence HIF-1α, PI3K/Akt, FOXM1, and other redox-sensitive signaling pathways, collectively promoting the metabolic flexibility required for tumor progression [204].

In addition to hypoxia and angiogenesis, NOX-derived ROS also interact with key tumor suppressor signaling pathways. Exogenous and NOX-generated ROS enhanced tumor growth specifically in PTEN-expressing (but not PTEN-deficient) glioblastoma cells, and NOX activity in response to radiation depended on the regulatory subunit p22, ensuring tumor cell survival while reducing the effectiveness of radiation therapy [205]. These findings support continued investigation of NOX4-targeted strategies in preclinical GB models relating to hypoxia, angiogenesis, PTEN/Akt signaling, and resistance to therapy in GB, and as a new target for redox-based therapeutic approaches that are now under investigation via computational and experimental testing of small-molecule NOX inhibitors for cancer drug development [102,163].

The role of NOX4 could be even more widespread because of its interaction with glioblastoma stem cells. Recent studies have demonstrated that patient-derived GSCs strongly express NOX4, and that TGF-β is able to induce its expression. NOX4 was upregulated upon TGF-β stimulation, producing more ROS in GSCs; NOX4 silencing or inhibition of its enzymatic activity decreased ROS [103,206]. Importantly, NOX4 activity was necessary for the TGF-β-induced proliferation, self-renewal, expression of stem-cell markers, and activation of antioxidant and metabolic responses mediated by NRF2 in GSCs [103,207]. The results extend the role of NOX4 beyond hypoxia and metabolism and indicate that NOX4 redox signaling might play a role in maintaining the stem-like phenotype that is believed to be responsible for the recurrence of glioblastoma.

The angiogenic effects of this network of signals are another significant link between NOX4 and tumorigenesis. The activation of HIF-1α leads to the production of pro-angiogenic factors such as VEGF and stimulates vascular remodeling and new vessel formation in hypoxic areas of the tumor [203,208]. NOX4 can indirectly affect angiogenesis by maintaining HIF-dependent transcription, as ROS generated by NOX4 can do the same [209]. Furthermore, not only the role of NOX4 in TGF-β signaling but also its involvement in GSC biology indicate that multiple independent components of the tumor microenvironment could converge on NOX4. Therefore, hypoxia, TGF-β signaling, ROS generation, stemness, metabolic reprogramming, angiogenesis, and therapeutic resistance could not be viewed as distinct mechanisms but instead as interconnected components of a larger network of signaling pathways regulated by NOX4 [103,163]. These findings provide a strong rationale for investigating NOX4 as a therapeutic target in glioblastoma. This raises the possibility that NOX4 inhibition could be more effective when combined with established GB treatments rather than used as a monotherapy. The findings of Hsieh et al., which demonstrated enhanced radiotherapy efficacy following NOX4 suppression in intracerebral GB models, provide direct preclinical support for this combination strategy [190].

An additional challenge concerns the translational development of NOX4 inhibitors. Although NOX4 has emerged as a promising therapeutic target in preclinical GB studies, clinical development of NOX4-targeted agents has largely occurred outside glioblastoma. For example, setanaxib (GKT137831), a first-in-class NOX1/4 inhibitor, has progressed into clinical trials for diseases including primary biliary cholangitis, kidney disease, and other fibrotic disorders, demonstrating that pharmacological inhibition of NOX1/4 is clinically feasible [210]. A randomized Phase II trial of setanaxib in primary biliary cholangitis provided human clinical experience with NOX1/4 inhibition, although the study did not establish efficacy in glioblastoma or other brain tumors [211,212]. Setanaxib has also been investigated in a Phase II oncology study in combination with pembrolizumab for recurrent or metastatic head and neck squamous cell carcinoma, further illustrating that NOX1/4 inhibition has progressed beyond purely preclinical research (ISRCTN63646763) [213]. However, these studies should not be interpreted as evidence of clinical efficacy against GB.

Accordingly, the current evidence supports a more cautious translational conclusion: NOX4 represents a promising but still experimentally unvalidated therapeutic target in glioblastoma, and clinical evidence specifically supporting NOX4 inhibition in GB remains lacking. This distinction is important because the promising results obtained with NOX4 inhibition in GB cell and animal models have not yet been translated into established clinical treatment. Future clinical development will require the identification of suitable selective inhibitors, confirmation of their ability to penetrate the blood–brain barrier, assessment of their pharmacodynamic effects within GB tissue, and determination of whether NOX4 inhibition can enhance the response to radiotherapy, TMZ, metabolic therapies, or immunotherapy.

Because hypoxia and NOX4 signaling are closely linked, several clinical strategies have instead focused on disrupting hypoxia-mediated metabolic adaptation. Dichloroacetate (DCA), a pyruvate dehydrogenase kinase inhibitor, promotes mitochondrial oxidative phosphorylation, increases mitochondrial ROS production, and partially reverses the Warburg phenotype [214,215]. Early clinical investigations of DCA in malignant brain tumors provided evidence that the drug is a small molecule capable of crossing the blood–brain barrier and is generally tolerable when administered orally, supporting its potential applicability to intracranial tumors (Figure 2) [216]. In a Phase I study involving 15 adults diagnosed with malignant brain tumors, including high-grade gliomas, chronic DCA administration was feasible, and no dose-limiting toxicities were observed during the initial treatment period; the main treatment-related concern was peripheral neuropathy, which occurred at low grade in a small number of patients [216]. However, the clinical evidence to date has not demonstrated substantial efficacy for DCA as a single agent, and its therapeutic benefit in glioblastoma remains insufficiently established. This has encouraged investigation of DCA as a metabolic sensitizer that could complement conventional treatment rather than replace it [216,217].

Similarly, metformin, an inhibitor of mitochondrial Complex I, has been evaluated in several Phase I and Phase II clinical trials in combination with radiotherapy and TMZ for newly diagnosed glioblastoma (Figure 2) [218]. Preclinical studies suggest that metformin can alter mitochondrial respiration and redox signaling [219,220]. However, the randomized phase II trial in GB found that metformin plus low-dose TMZ was well tolerated but did not significantly improve progression-free survival or overall survival compared with TMZ alone. Although clinical benefits have been limited, these studies confirmed the safety and feasibility of targeting tumor metabolism alongside conventional therapy and have encouraged further investigation of combination approaches [218,219].

A direct approach to inhibiting NOX4 is to target the tumor microenvironment where it is activated, namely, tumor hypoxia. Hypoxia-activated prodrugs (HAPs) are cytotoxic effectors that are selectively released under low oxygen. The extensively studied HAP, Evofosfamide (TH-302), is a compound that breaks down under hypoxic conditions and releases a DNA-crosslinking agent under reductase-dependent conditions [221,222]. It was evaluated in pancreatic cancer in two Phase I trials (NCT01833546, NCT02047500), which resulted in a Phase III trial (NCT01746979) that concluded with a lack of a primary survival benefit; in addition, a parallel soft-tissue sarcoma trial (NCT01440088) was completed, and a Phase II NSCLC trial (NCT02093962) was stopped for futility [223,224,225]. In more recent years, however, clinical research has begun to examine and exploit the spatial variability of hypoxia rather than consider hypoxia as a homogeneous tumor property. An ongoing prospective study (NCT05500612) is testing the performance of oxygen-enhanced MRI and BOLD MRI in detecting hypoxic regions in GB and is assessing the potential to use these imaging data to inform selective radiation dose escalation in biologically aggressive tumor subregions [226].

Overall, these studies indicate that targeting hypoxic GB will require strategies to deal with the spatial and temporal diversity of GB, in addition to eliminating the hypoxic cells. The addition of hypoxia-directed or hypoxia-guided interventions—radiotherapy, metabolic inhibitors, or redox adaptation inhibitors—to tumor treatment may disrupt the balance of hypoxia-directed or hypoxia-guided NOX4–ROS–HIF-1α signaling and enhance tumor sensitivity to the oxidative and genotoxic effects of radiation therapy. However, this therapeutic paradigm is still largely being investigated, and the few clinical responses reported with hypoxia-activated approaches highlight the need for better biomarkers that could identify the hypoxic, redox-adapted tumor compartments that will best respond to such therapeutic approaches.

### 5.4. Oxidative Stress and BBB Penetration: Towards Multimodal Therapeutic Approaches for Glioblastoma

Although overall survival for glioblastoma is only 15–20 months, the standard of care involves maximal safe surgical resection, concurrent radiotherapy, and TMZ-based chemotherapy [8,227]. Therapeutic efficacy is known to be associated with both radiotherapy and chemotherapy, and there is a good correlation between therapeutic efficacy and the induction of oxidative stress. Because both radiotherapy and TMZ can perturb tumor redox homeostasis, treatment efficacy is closely linked to whether GB cells can tolerate or exploit the resulting oxidative stress [142,144,228,229].

This has led to a shift in focus to the development of adjuvant therapies that selectively enhance ROS-mediated killing and overcome antioxidant-mediated resistance. Tumor Treating Fields is an FDA-approved adjunctive therapy for newly diagnosed GB that disrupts the assembly of the mitotic spindle and cytokinesis, sensitizes glioma cells to TMZ and mitochondrial dysfunction, and leads to intracellular ROS accumulation [230].

Concurrently, there have been a growing number of targeted drugs, such as tyrosine-kinase inhibitors and anti-angiogenic agents (bevacizumab), that are being explored for their potential to target oncogenic signaling pathways and modulate cellular redox balance, but their clinical efficacy is limited by tumor heterogeneity, adaptive signaling, and poor drug penetration of the blood–brain barrier [227,231]. The blood–brain barrier (BBB) is a major challenge for successful GB therapy, especially for molecularly targeted drugs such as kinase inhibitors. The BBB remains a major translational barrier because efflux transporters such as ABCB1 and ABCG2 can prevent otherwise potent agents from reaching therapeutic concentrations in infiltrative tumor regions [231,232]. The BBB is composed of unique and specialized brain endothelial cells surrounded by tight junction proteins, pericytes, astrocytic axon terminals, and the basement membrane, and is tightly involved in controlling molecular transport into the central nervous system [232]. Besides its physical barrier role, the BBB also exhibits ATP-binding cassette (ABC) efflux transporters such as P-glycoprotein (ABCB1) and breast cancer resistance protein (BCRP/ABCG2), which actively transport many kinase inhibitors from endothelial cells back into circulation, significantly limiting their accumulation within the brain [233]. This led to the development of many first-generation tyrosine kinase inhibitors (TKIs) with high preclinical potency that have proven less effective in the clinic because they cannot reach therapeutic levels in infiltrative tumor areas [231,233].

To overcome these limitations, recent therapeutic strategies have focused on optimizing the physicochemical properties of kinase inhibitors by reducing molecular weight and polar surface area, increasing lipophilicity, and minimizing recognition by efflux transporters to improve BBB penetration [231]. In addition, novel delivery strategies such as nanoparticle-based drug delivery, receptor-mediated transcytosis, focused ultrasound-mediated transient BBB opening, convection-enhanced delivery, and biodegradable intracranial implants have demonstrated significant potential to enhance drug delivery and therapeutic efficacy in the brain [232,234]. All of these results suggest a need for innovative delivery systems and multimodal therapies that can be incorporated into BBB-penetrant kinase inhibitors to efficiently treat GB, both in the highly infiltrated margins and the protected core of the tumor [231].

On the other hand, naturally derived compounds that modulate redox processes and nanotechnology drug delivery systems have recently been found to be effective adjuvants. A phyto-mycotherapeutic formulation, enriched with bioactive extracts from medicinal mushrooms, significantly inhibited the growth of U251 glioblastoma cells by modulating oxidative stress through pro-oxidative effects, reduction in mitochondrial function, promotion of apoptosis, and enhancement of the pro-death effects of TMZ, indicating that modulation of oxidative stress could improve the effects of conventional therapy [235,236]. Similarly, nanoliposomes containing a combination of cannabidiol (CBD) and celecoxib were found to simultaneously modulate the level of oxidative stress and inflammatory signaling in experimental high-grade gliomas, with a potential for boosting the level of ROS, reducing the activity of NF-κB and Wnt/β-catenin signaling, activating NRF2-dependent antioxidant signaling, and inducing cell death by apoptosis in tumor cells, thus improving the therapeutic potential of the combination therapy [237].

Other experiments have further confirmed that CDN activates the ERK signaling pathway, induces endoplasmic reticulum stress, autophagy, ferroptosis, glutathione depletion, and mitochondrial dysfunction in a ROS-dependent manner, suggesting that ROS is a major mediator of its antitumor activity [238]. Taken together, these results indicate that oxidative stress is a common mechanism of action for both established and novel adjuvant treatments. In contrast to ROS as a mere by-product of treatment, ROS is now recognized as a key therapeutic target, and its controlled modulation can improve treatment effectiveness, overcome resistance, and provide a rationale for the development of multimodal redox-based therapeutic strategies for GB [174,229].

## 6. Conclusions

Oxidative stress plays a central and multifaceted role in the pathogenesis and progression of glioblastoma, influencing tumor initiation, metabolic reprogramming, and resistance to therapy. The balance between ROS production and antioxidant defenses not only drives genomic instability and oncogenic signaling but also enables tumor cells to adapt to harsh microenvironmental conditions. Key oxidative stress-related biomarkers, including mitochondrial enzymes, NADPH oxidases, and antioxidant systems, highlight the complexity and dual nature of redox regulation in GB.

Importantly, GB cells’ ability to exploit antioxidant pathways, particularly through mechanisms such as Nrf2 activation, contributes significantly to resistance to radiotherapy and chemotherapy. These insights underscore the potential of targeting redox homeostasis as a therapeutic strategy. Future research should focus on integrating oxidative stress biomarkers into clinical practice and developing approaches that selectively disrupt tumor redox balance while preserving normal cellular function, ultimately improving patient outcomes.

## Data Availability

No new data were created or analyzed in this study. Data sharing does not apply to this article.
